# Sertraline as a Multi-Target Modulator of AChE, COX-2, BACE-1, and GSK-3β: Computational and In Vivo Studies

**DOI:** 10.3390/molecules29225354

**Published:** 2024-11-14

**Authors:** Minhajul Arfeen, Vasudevan Mani

**Affiliations:** 1 Department of Medicinal Chemistry and Pharmacognosy, College of Pharmacy, Qassim University, Buraydah 51452, Saudi Arabia; 2 Department of Pharmacology and Toxicology, College of Pharmacy, Qassim University, Buraydah 51452, Saudi Arabia; v.samy@qu.edu.sa

**Keywords:** sertraline, machine learning, molecular docking simulation, virtual screening, Alzheimer’s diseases, diabetes, neurodegenerative disorder

## Abstract

Alzheimer’s disease (AD) is a neurodegenerative disorder associated with the dysregulation of several key enzymes, including acetylcholinesterase (AChE), cyclooxygenase-2 (COX-2), glycogen synthase kinase 3β (GSK-3β), β-site amyloid precursor protein cleaving enzyme 1 (BACE-1), and caspase-3. In this study, machine learning algorithms such as Random Forest (RF), Gradient Boost (GB), and Extreme Gradient Boost (XGB) were employed to screen US-FDA approved drugs from the ZINC15 database to identify potential dual inhibitors of COX-2 and AChE. The models were trained using molecules obtained from the ChEMBL database, with 5039 molecules for AChE and 3689 molecules for COX-2. Specifically, 1248 and 3791 molecules were classified as active and inactive for AChE, respectively, while 858 and 2831 molecules were classified as active and inactive for COX-2. The three machine learning models achieved prediction accuracies ranging from 92% to 95% for both AChE and COX-2. Virtual screening of US-FDA drugs from the ZINC15 database identified sertraline (SETL) as a potential dual inhibitor of AChE and COX-2. Further docking studies of SETL in the active sites of AChE and COX-2, as well as BACE-1, GSK-3β, and caspase-3, revealed strong binding affinities for all five proteins. In vivo validation was conducted using a lipopolysaccharide (LPS)-induced rat model pretreated with SETL for 30 days. The results demonstrated a significant decrease in the levels of AChE (*p* < 0.001), BACE-1 (*p* < 0.01), GSK-3β (*p* < 0.05), and COX-2 (*p* < 0.05). Additionally, the downstream effects were evaluated, showing significant decreases in the apoptosis marker caspase-3 (*p* < 0.05) and the oxidative stress marker malondialdehyde (MDA) (*p* < 0.001), indicating that SETL is clinically localized in its effectiveness, mitigating both enzymatic activity and the associated pathological changes of cognitive impairment and AD.

## 1. Introduction

Memory impairment and dementia, including Alzheimer’s disease (AD), present major global health and economic challenges. As of 2024, over 10% of Americans aged 65 and older suffer from cognitive impairment, with 15–22% experiencing mild cases. Worldwide, dementia cases are expected to reach 139 million by 2050 [1]. The economic impact is significant: global costs hit USD 1 trillion in 2019 and are projected to more than double by 2050. In the U.S., AD care costs exceeded USD 345 billion in 2023 and may surpass USD 1 trillion by mid-century. AD was also the seventh leading cause of death in the U.S. in 2022. Healthcare costs for dementia patients are nearly three times higher than for those without the condition [2,3].

AD is a multifactorial neurodegenerative disorder characterized by the dysregulation of various macromolecules. Acetylcholinesterase (AChE) is a well-established molecular target for AD, and AChE inhibitors are among the first-line treatments for its symptoms. Common AChE inhibitors include donepezil, rivastigmine, and galantamine. Donepezil is used to treat mild, moderate, and severe forms of AD, while rivastigmine and galantamine are prescribed for mild to moderate cases [4]. In addition to AChE, β-secretase (BACE-1) is another validated target in AD drug discovery. However, BACE-1 inhibitors have not yielded clinical benefits due to their association with severe side effects, including cognitive worsening, liver toxicity, and other safety concerns. For example, verubecestat (MK8931), one of the most advanced BACE-1 inhibitors, failed in clinical trials due to a lack of cognitive benefits [5]. Lanabecestat (AZD3293) failed because of poor efficacy; Atabecestat (JNJ-54861911) due to liver toxicity; and Umibecestat (CNP520) showed cognitive decline, contrary to the intended effect [6,7,8].

Glycogen synthase kinase-3β (GSK-3β), an isoform of GSK-3β, is well-documented for its role in the pathological processes of Alzheimer’s disease (AD) [9]. GSK-3β plays a central role in tau hyperphosphorylation, Aβ production, and synaptic dysfunction in AD [10,11,12]. It also plays a crucial role in neuroinflammation. Over-activation of GSK-3β is associated with increased levels of proinflammatory cytokines, contributing to the neuroinflammation seen in AD [13,14]. Despite being a promising drug target, no GSK-3β inhibitors have achieved clinical success. For instance, tideglusib and lithium, both GSK-3β inhibitors tested in clinical trials, failed to show significant cognitive benefits. Neuroinflammation, a well-established factor in disease progression, involves the enzyme COX-2, which has gained attention as a potential target for AD treatment due to its role in inflammation [15,16]. The upregulation of COX-2 in the brains of AD patients contributes to tau hyperphosphorylation, amyloid-beta production, and increased proinflammatory cytokines and oxidative stress, leading to neuronal damage and cognitive decline [17]. However, selective COX-2 inhibitors such as celecoxib, tested in clinical trials, have shown no success, suggesting that inhibiting COX-2 alone may be insufficient to address the complex pathology of AD [18].

**Figure 1:**
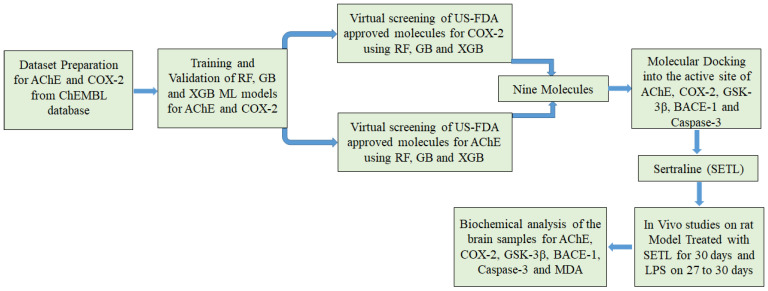
Workflow utilized in this report.

Machine learning models (ML), under the umbrella of artificial intelligence, have transformed the drug discovery field by leveraging data-driven insights to uncover novel therapeutic targets and predict drug interactions. This approach streamlines the drug development pipeline, improves predictive accuracy, and accelerates the identification of promising compounds [19]. In this work, the ML models RF, GB, and XGB were employed to screen US-FDA approved drugs available from the ZINC15 database to identify potential dual inhibitors for AChE and COX-2. The virtual screening results from ML model XGB identified SETL as a potential dual inhibitor of AChE and COX-2. Following virtual screening, molecular docking was conducted to predict the binding affinity of SETL for GSK-3β, BACE-1, and caspase-3 in addition to AChE and COX-2, thus further broadening the scope of our multi-target exploration. The results from molecular docking indicated substantial binding affinity for the target proteins comparable to co-crystallized ligand. SETL was subsequently evaluated using an LPS-induced rat model to assess its efficacy against the aforementioned target proteins AChE, COX-2, GSK-3β, BACE-1, and caspase-3 as well as MDA, an oxidative stress marker. The results from the in vivo study demonstrated significant reductions in the levels of target enzymes and MDA, indicating SETL’s potential therapeutic efficacy. Figure 1 shows the workflow utilized in this report. Notably, no ML models have been reported for the identification of a dual inhibitor against AChE and COX-2. Furthermore, SETL has been documented to modulate AChE, GSK-3β in the platelets, caspase-3 in the cancer cells, and MDA. SETL has not been reported to modulate COX-2 and BACE-1.

## 2. Results

### 2.1. Data Preparation

The obtained dataset was refined using the following criteria: (i) only compounds with a binding assay type of “B” were included, (ii) compounds without well-defined IC50 values were excluded, (iii) duplicate entries were removed, (iv) compounds lacking canonical SMILES were discarded, and (v) IC50 units were standardized by converting them to nM. Additionally, the IC50 values were normalized by converting them to pIC50 values. The final refinement yielded 5039 molecules for AChE and 3689 molecules for COX-2. Compounds with pIC50 values above seven were considered active, while those below seven were deemed inactive. Therefore, the final number of molecules obtained for AChE and COX-2 were segregated into active and inactive categories. The categorization resulted in 1248 active molecules and 3791 inactive molecules for AChE. Similarly, the categorization for COX-2 showed 858 active and 2831 inactive molecules. Finally, class balancing was performed to obtain datasets with 7582 and 5662 entries for AChE and COX-2, respectively. The final numbers after class balancing were then divided into training and test sets at a ratio of 80:20.

### 2.2. Generation of Molecular Descriptors

Molecular descriptors for the refined dataset were generated using the RDKit toolkit. A total of 210 molecular descriptors or features were generated. Because every feature does not contribute to defining the biological properties of the molecules, the features with a value of zero were excluded in order to reduce the computational time.

### 2.3. Chemical Space Analysis

**Figure 2:**
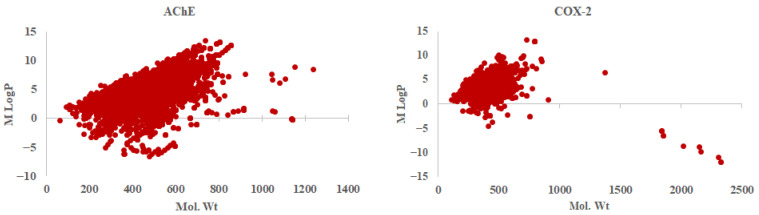
Chemical space analysis of training and test sets. The molecular weight and MlogP define the chemical space considered in this study.

Chemical space refers to the vast, multidimensional set of all possible chemical compounds, encompassing both known and hypothetical molecules. Understanding how variations in chemical structure influence biological or chemical properties (SAR) is fundamental to chemical research. Machine learning models trained on diverse chemical spaces can predict novel compounds, facilitating the rational design of molecules with specific, desired properties. Accurately representing molecular diversity within training datasets enables models to generalize more effectively and make more precise predictions about previously unseen compounds. In the present study, the chemical space analysis was carried out by plotting logP against molecular weight. For AChE, the LogP value varied in the range of ~−6 to −14, and molecular weight varied in the range of ~60 to 1250 Da. Similarly, for COX-2 the logP value and molecular weight varied from ~−12 to 13 and ~108 to 2300 Da, respectively. Figure 2 shows the chemical space for the molecules of the training set as well as the test set for both AChE and COX-2.

### 2.4. Model Generation and Validation

Table 1 shows the performance metrics of the three machine learning models RF, GB, 0and XGB evaluated on the datasets of AChE and COX-2. For the COX dataset, RF achieved a sensitivity of 0.94, specificity of 0.91, and a balanced F1-Score of 0.93, with an accuracy of 0.92. XGB performed slightly better in terms of sensitivity, reaching 0.96, but at the cost of lower specificity (0.87), while maintaining a similar F1-Score and accuracy (0.93 and 0.92, respectively). GB had a sensitivity of 0.93 and a specificity of 0.88, with slightly lower precision and F1-Score values (0.89 and 0.92). For the AChE dataset, RF once again showed strong performance, with a sensitivity of 0.96 and a specificity of 0.933, resulting in an accuracy of 0.95 and an F1-Score of 0.93. XGBoost showed similar high sensitivity (0.97) but with a slightly lower specificity (0.92), yielding an accuracy of 0.94. GB exhibited comparable results with a sensitivity of 0.97 and specificity of 0.92. Overall, both RF and XGB demonstrated high and balanced sensitivity and specificity, making them robust models for both datasets. XGB marginally outperformed RF in sensitivity, while RF offered slightly better precision. Both models are reliable choices for classification tasks, striking a strong balance between false positives and false negatives. Therefore, both RF forest and XGB were chosen to screen the US-FDA approved drugs present in the ZINC15 database. It is pertinent to mention that molecules from the ZINC15 database were screened using COX-2-trained models, followed by AChE-trained models. The virtual screening with the RF model did not identify any potential dual inhibitors of COX-2 and AChE, whereas XGB successfully identified SETL [20]. Table S1 (Supplementary Materials) shows the settings/hyperparameters used to generate the performance metrics of the three machine learning models used in this study.

### 2.5. Molecular Docking Simulation

**Figure 3:**
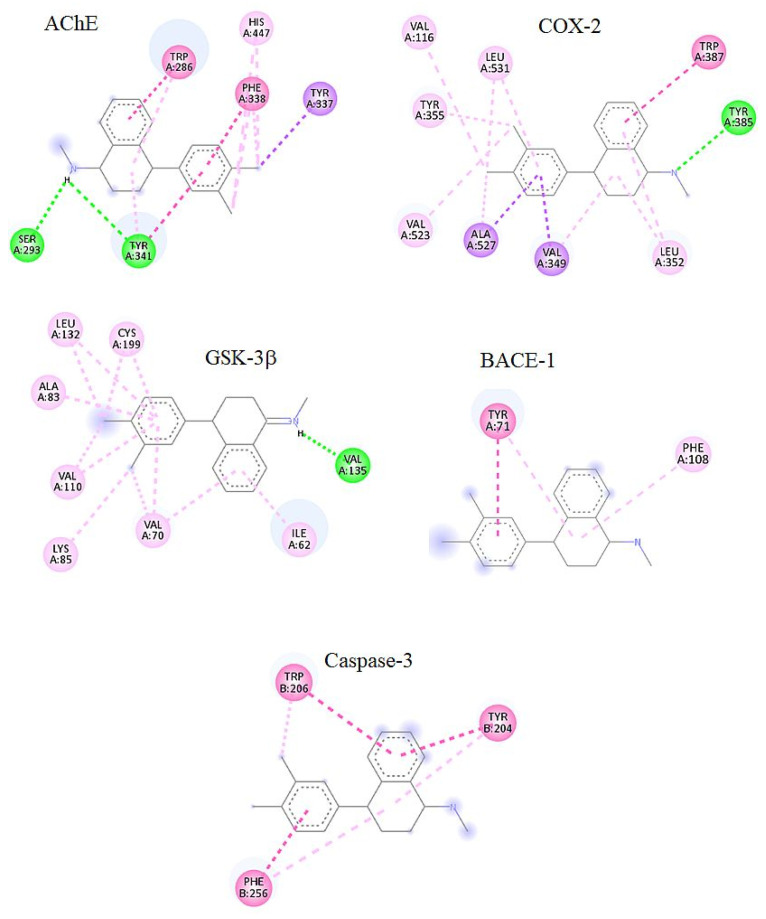
Hydrogen (green) and hydrophobic interactions (pink and purple) of SETL in the active sites of AChE, COX-2, GSK-3β, BACE-1, and caspase-3.

After identifying SETL as a potential dual inhibitor of AChE and COX-2 using machine learning models, molecular docking was performed to evaluate their molecular interactions. SETL exhibited a significant binding score for both AChE and COX-2, comparable to co-crystallized ligands. The binding scores for SETL against AChE and COX-2 were approximately 10.5 and 8.6 kcal/mol, respectively (Table S2, Supplementary Materials). The binding affinities for the co-crystallized ligands of AChE and COX-2 were approximately 12.1 and 9.1 kcal/mol, respectively. In addition to AChE and COX-2, molecular docking was also performed on other molecular targets linked to cognitive impairment, including GSK-3β, BACE-1, and caspase-3. The binding affinities of SETL for GSK-3β, BACE-1, and caspase-3 were 9.2, 7.2, and 8.4 kcal/mol, respectively. The calculated binding affinities of the co-crystallized ligands for GSK-3β, BACE-1, and caspase-3 were 9.2, 7.6, and 8.8 kcal/mol, respectively. Further analysis of the top three low-energy conformations revealed that the lowest energy conformation of SETL displayed molecular interactions with key residues in all docked complexes. The docked complex of SETL with AChE formed hydrogen bonds with Ser293 and Tyr341, while hydrophobic interactions were observed with Tyr337, Trp286, Phe338, Tyr341, and His447. In the docked complex of SETL with COX-2, hydrogen bonds were observed with Tyr385, and hydrophobic interactions occurred with Lys83, Val89, Tyr115, Val116, Arg120, Phe470, and Met471. For the docked complex of SETL with GSK-3β, hydrogen bonding was observed with Val135, while hydrophobic interactions were noted with Ile62, Val70, Ala83, Lys85, Val110, Leu132, and Cys199. In the docked complexes of BACE-1 and caspase-3, no polar interactions were observed. However, hydrophobic interactions were noted. For BACE-1, these occurred with T’yr71 and Phe108, and for caspase-3, these occurred with Tyr204, Trp206, and Phe256. Figure 3 shows the binding mode of SETL in the active site of AChE, COX-2, GSK-3β, BACE-1, and caspase-3. Figures S1 and S2 (Supplementary Materials) show the overlapping of the docked ligand and SETL with the co-crystallized ligand, respectively. It should be noted that the abovementioned interactions of SETL with AChE, COX-2, GSK-3β, BACE-1, and caspase-3 are similar to the interactions observed with co-crystallized ligand (Figure S3).

### 2.6. Biochemical Analysis

**Figure 4:**
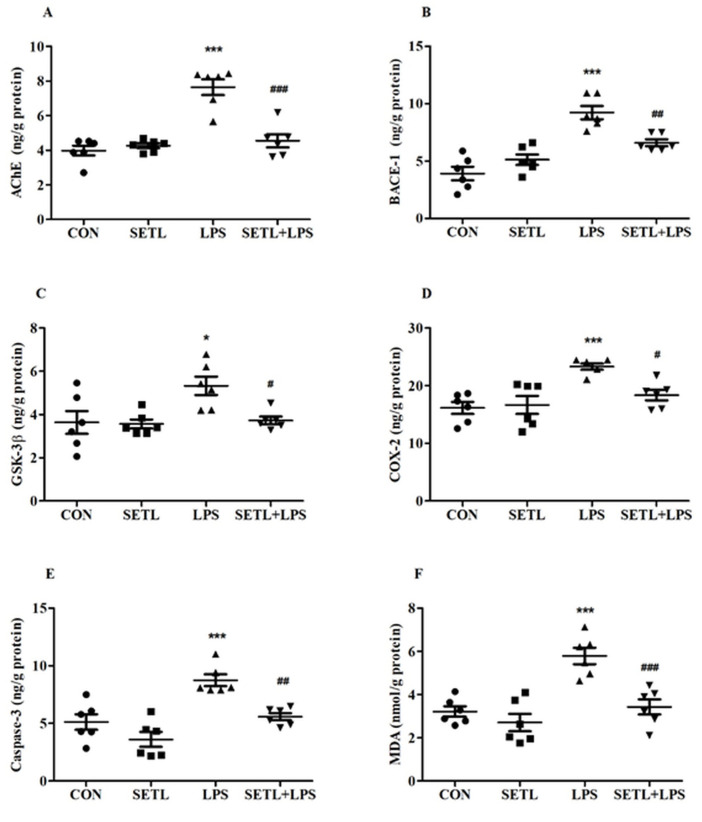
Effect of sertraline (SETL) on (**A**) AChE, (**B**) BACE-1, (**C**) GSK-3β, (**D**) COX-2, (**E**) caspase-3, and (**F**) MDA in LPS-induced neurotoxicity rats. The results are expressed as mean ± SEM, with a sample size of n = 6. One-way ANOVA followed by Tukey–Kramer multiple comparisons test. * *p* < 0.05 and *** *p* < 0.001 as compared to the control group; ^#^ *p* < 0.05, ^##^ *p* < 0.01, and ^###^ *p* < 0.001 as compared to the LPS group.

The results show that SETL significantly modulates biomarkers related to neurodegeneration, inflammation, apoptosis, and oxidative stress in LPS-induced neurotoxicity rats. The biomarkers studied include AChE, BACE-1, GSK-3β, COX-2, caspase-3, and MDA. Statistical analysis using one-way ANOVA with Tukey–Kramer multiple comparisons revealed significant differences between the treatment groups. SETL+LPS treatment led to significant reductions in these biomarkers compared to the group treated with LPS only, underscoring its potential neuroprotective role. For AChE, which degrades acetylcholine (ACh), LPS treatment significantly increased AChE levels compared to the control group (*p* < 0.001). SETL administration, however, significantly reduced AChE levels compared to the LPS group (*p* < 0.001) and restored them to close to control levels. This suggests that SETL can mitigate LPS-induced cholinergic dysfunction, potentially preserving cognitive function by preventing excessive ACh degradation. With regard to BACE-1, the enzyme responsible for amyloid production, LPS treatment caused a significant increase in BACE-1 levels compared to the control group (*p* < 0.001). SETL significantly lowered BACE-1 levels compared to the LPS group (*p* < 0.01), indicating that SETL may reduce β-amyloid production, a hallmark of AD. Importantly, SETL-treated rats had BACE-1 levels approaching those seen in the control group, suggesting it may effectively counteract the amyloidogenic pathway activated by LPS. GSK-3β, which plays a role in both tau phosphorylation and inflammation, was significantly increased in the LPS group compared to the control group (*p* < 0.05). SETL treatment led to a significant reduction in GSK-3β levels compared to the LPS group (*p* < 0.05), with GSK-3β approaching control levels. This suggests that SETL may inhibit both tau hyperphosphorylation and the inflammatory response triggered by LPS, which are key factors in AD pathology. In the case of COX-2, an enzyme that promotes inflammation, LPS treatment led to significantly elevated levels compared to the control group (*p* < 0.001). SETL reduced COX-2 levels in the LPS-treated group (*p* < 0.05), bringing them closer to the levels observed in the control group. This finding underscores SETL’s anti-inflammatory effects, which may help reduce neuroinflammation, a critical contributor to neurodegenerative diseases. For caspase-3, which plays a major role in apoptosis, LPS treatment caused a significant increase in its levels compared to the control (*p* < 0.001). SETL administration resulted in a marked reduction in caspase-3 levels compared to the LPS group (*p* < 0.01), nearing control levels. This suggests that SETL may prevent LPS-induced neuronal apoptosis, protecting against cell death and helping to maintain neuronal integrity. Lastly, MDA, a marker of oxidative stress, was significantly elevated in the LPS group compared to the control (*p* < 0.001). SETL treatment significantly reduced MDA levels (*p* < 0.001) compared to the LPS group, approaching control group levels. This indicates that SETL has strong antioxidant properties, reducing oxidative damage to neurons caused by LPS-induced stress. In conclusion, SETL significantly reduces AChE, BACE-1, GSK-3β, COX-2, caspase-3, and MDA levels in LPS-induced neurotoxicity. The reductions in these biomarkers not only bring their levels closer to those of the control group but also highlight SETL’s potential to target multiple pathological mechanisms in neurodegenerative diseases such as AD. Figure 4 shows the results from the biochemical analysis.

## 3. Discussion

The “one drug, one target” concept has been a cornerstone of drug discovery for several decades [21]. While this strategy has led to the development of numerous successful medications, it also presents limitations when dealing with complex diseases such as AD that involve multiple interconnected pathways. As a result, the traditional single-target approach is often insufficient for treating complex diseases. This has driven a shift toward developing multi-target drugs (MTDLs) that can simultaneously modulate multiple pathways or targets. By doing so, MTDLs leverage the inherent robustness of biological systems, addressing not only the primary disease mechanisms but also compensatory pathways that may contribute to disease progression [22,23,24]. In this work, three ML models were used to virtually screen the US-FDA approved drugs listed in the ZINC15 database. Further, molecular docking was performed against GSK-3β, BACE-1, and caspase-3. Taken together, the results from ML and molecular docking underlined the potential of SETL as an MTDL for AChE, COX-2, GSK-3β, and BACE-1. Further, the potential of SETL as an MTDL was evaluated using the brain samples from rats subjected to LPS-induced neurotoxicity. This study utilized four systemic injections of LPS (1 mg/kg, i.p.) to create a model for neuroinflammation-related diseases. Previous reports have shown that LPS triggers both neuroinflammation and neurodegeneration in animal models [25,26,27,28,29,30]. In addition to the biochemical analyses of AChE, COX-2, GSK-3β, and BACE-1, the brain samples were also analyzed for caspase-3 and MDA. Our results from this study demonstrate the potential of SETL to alleviate the effects on the specified molecular targets and reduce oxidative stress. It is pertinent to mention that the potential neuroprotective role of SETL, particularly concerning neuroinflammation and associated pathways, has been minimally explored and remains largely underreported in the existing literature.

Machine learning models are enhancing drug discovery by leveraging data-driven insights to accelerate target identification, predict drug interactions, and streamline the drug development process [31,32,33]. This study utilizes compounds from the ChEMBL database, focusing on AChE and COX-2, specifically targeting human protein inhibitors using UniProt IDs P22303 and P35354. Molecular descriptors, including physicochemical properties such as molecular weight, LogP, TPSA, and hydrogen bond donors/acceptors, were calculated using RDKit. The three machine learning algorithms RF, GB, and XGB were employed to identify dual inhibitors, each with distinct strengths. RF constructs multiple decision trees for robust predictions, GB iteratively corrects errors, and XGB optimizes GB for improved speed and accuracy [34]. Model evaluation was conducted using sensitivity, specificity, precision, overall accuracy, and F1 score, providing a comprehensive assessment of their effectiveness in identifying dual inhibitors within complex datasets. The dataset was refined to include only compounds with binding assay type “B”, excluding those without clear IC50 values or canonical SMILES, and standardizing IC50 values to nM. The compounds with an IC50 value of 100 nM (pIC50 ≥ 7) or less were considered as potent, while the compounds with an IC50 value greater than 100 nM (pIC50 ≤ 7) were considered less potent. Based on the threshold, the compounds were defined as active and inactive resulting in 1248 active and 3791 inactive AChE molecules, along with 858 active and 2831 inactive COX-2 molecules. After class balancing, the datasets comprised 7582 entries for AChE and 5662 for COX-2, split into training and test sets at an 80:20 ratio. The 80:20 split between the training and test sets ensures that the model is trained on a substantial portion of the data while leaving enough data for unbiased evaluation. The 80% training set allows the model to learn from a robust dataset, while the 20% test set provides sufficient data to assess the model’s performance without overfitting. A total of 210 molecular descriptors were generated for the dataset, with features with a zero value being excluded to streamline the analysis. Chemical space analysis, plotting logP against molecular weight, revealed substantial diversity in the training and test sets. Ultimately, RF and XGB demonstrated superior performance, with XGB achieving slightly higher sensitivity. Both models were applied to screen FDA-approved drugs from the ZINC15 database, with XGB successfully identifying potential dual inhibitors, while RF did not identify any.

Molecular docking is a vital tool for predicting both the binding affinity and orientation of compounds to target proteins, aiding in the discovery of potential therapeutic candidates [35]. In this study, molecular docking was employed to assess the binding affinity of SETL within the active sites of AChE and COX-2. Additionally, docking simulations were performed on GSK-3β, BACE-1, and caspase-3. Our results indicate that SETL shows strong binding affinities comparable to those of co-crystallized ligands, suggesting its potential to address cognitive impairment by targeting multiple pathways. A detailed binding mode analysis was conducted on the three top-ranked low-energy conformations, and the lowest-energy conformation was selected for further study as it exhibited key interactions with critical residues. In the AChE-SETL complex, hydrogen bonds and hydrophobic interactions were observed with Tyr341, while Trp286 and His447 participated only in hydrophobic interactions. Notably, Tyr341 and Trp286 are part of the peripheral anionic site, which is essential for binding inhibitors and substrates, whereas His447 plays a pivotal role in ACh hydrolysis [36,37,38,39]. For COX-2, SETL formed a hydrogen bond with Tyr385, situated at the bend of the L-shaped hydrophobic tunnel, and engaged in hydrophobic interactions with Arg120, a key gatekeeper residue. The hydrogen bonds with Tyr385 and Arg120 are similar to those found in NSAIDs, such as phenylacetic acid and benzoic acid derivatives. Furthermore, the hydrophobic tunnel facilitates the access of arachidonic acid to the enzyme’s oxygenation site [40]. In the GSK-3β complex, SETL formed a hydrogen bond with Val135, a crucial residue for molecular recognition. Hydrophobic interactions were observed with residues Ile62, Val70, Ala83, Lys85, Val110, Leu132, and Cys199, all of which are critical for maintaining the potency of small-molecule inhibitors [9,41,42]. In the BACE-1 docking model, SETL formed hydrophobic contacts with Tyr71 and Phe108, which play essential roles in stabilizing inhibitor binding [43]. For caspase-3, SETL established hydrophobic interactions with Tyr204, Trp206, and Phe256—residues unique to caspase-3 that are significant for selective inhibitor binding [44].

AChE plays a pivotal role in the degradation of ACh, a neurotransmitter involved in memory and learning. In neurodegenerative diseases such as AD, elevated AChE levels exacerbate cholinergic dysfunction, leading to impaired cognitive function. Our results indicate that SETL significantly reduced AChE activity in LPS-treated rats, as evidenced by the marked *p*-values. This reduction suggests that SETL may protect against neurotoxicity by preserving cholinergic signaling. By decreasing ACh breakdown, SETL has the potential to improve cognitive outcomes, making it a potential candidate for treating conditions characterized by cholinergic deficits. This effect is particularly relevant in AD, where ACh depletion is a hallmark, and therapies that enhance cholinergic function, such as AChE inhibitors, are a standard treatment approach [4]. Our findings are consistent with previous reports, reinforcing the idea that SETL has the ability to modulate AChE activity and thus may contribute to its potential neuroprotective effects, extending beyond its well-established function as a selective serotonin reuptake inhibitor (SSRI) [45,46].

BACE-1 is a critical enzyme in the amyloidogenic pathway, responsible for the cleavage of amyloid precursor protein (APP) and the subsequent formation of β-amyloid peptides, which aggregate to form amyloid plaques in AD. Elevated BACE-1 activity is directly linked to increased β-amyloid production, a central feature of AD pathology. The results demonstrated a significant reduction in BACE-1 levels in the SETL-treated group compared to the LPS group. This finding is particularly promising, as it suggests that SETL may reduce amyloid plaque formation by inhibiting the early stages of β-amyloid production. A marked reduction in BACE-1 activity could slow or even prevent the progression of amyloid-related neurodegeneration, positioning SETL as a potential disease-modifying agent in AD treatment. The implications of this are far-reaching, given the central role that amyloid plaques play in the neurodegenerative cascade of AD. Targeting BACE-1 has been a focus of therapeutic development in AD research, and the observation that SETL can influence this pathway warrants further investigation. Notably, the literature search did not provide evidence of SETL modulating BACE-1 directly. However, previous studies have reported a reduction in β-amyloid levels in response to SSRI treatments [47,48,49].

GSK-3β is another critical player in neurodegenerative diseases, particularly due to its role in phosphorylating tau protein, leading to the formation of neurofibrillary tangles, a hallmark of AD [12]. GSK-3β also plays a role in inflammation [50], oxidative stress [51], and apoptosis, making it a key target in neuroprotective strategies [52]. The significant reduction in GSK-3β levels observed in the SETL-treated group suggests that the drug may exert protective effects by modulating tau pathology, reducing neurofibrillary tangle formation, and attenuating inflammatory signaling pathways. The inhibition of GSK-3β by SETL could also have implications beyond AD, as this enzyme is involved in multiple cellular processes related to inflammation, cell death, and synaptic plasticity. The reduction in GSK-3β levels points to SETL’s potential to modulate both inflammatory and tau-related pathological processes, thereby providing a dual mechanism of neuroprotection. This is critical, as AD pathology involves both amyloid plaques and tau tangles, and therapies that target both are likely to be more effective than those addressing a single pathway. Our results are in line with previously reported studies, where other SSRIs have been demonstrated to have inhibitory effects on GSK-3β from brain samples [53,54,55,56]. However, a study from the samples of platelets demonstrated an increase in the total expression of GSK-3β along with the active form upon long-term treatment with SETL [57].

COX-2 is an enzyme that catalyzes the formation of proinflammatory prostaglandins and is upregulated in various neuroinflammatory conditions, including AD [58,59]. In the context of LPS-induced neurotoxicity, COX-2 levels are typically elevated, reflecting a heightened inflammatory response. SETL’s ability to significantly reduce COX-2 levels suggests that it has potent anti-inflammatory properties, which could be crucial in mitigating the inflammatory component of neurodegeneration. The reduction in COX-2 implies that SETL might dampen the production of inflammatory mediators, reducing neuroinflammation and protecting neurons from inflammatory damage. Neuroinflammation is a key driver of neurodegenerative processes, and interventions that can reduce inflammation are likely to have broad therapeutic potential [60,61]. The significant decrease in COX-2 levels observed in this study suggests that SETL may attenuate the inflammatory response in neurodegenerative diseases, providing another mechanism by which it could protect against neurotoxicity.

Caspase-3 is a central executor of apoptosis, and elevated caspase-3 activity is a hallmark of neurodegenerative diseases where increased neuronal cell death is observed [62]. In the LPS-induced neurotoxicity model, caspase-3 levels were significantly elevated, indicating enhanced apoptotic activity. SETL’s ability to reduce caspase-3 levels suggests that it may protect neurons by inhibiting apoptosis. This is particularly important in the context of neurodegenerative diseases, where excessive neuronal loss is a key pathological feature. By reducing caspase-3 levels, SETL may help preserve neuronal integrity and function, thereby preventing the progressive loss of neurons that characterizes conditions such as AD. The marked reduction in caspase-3 levels also suggests that SETL could reduce overall brain cell death, further highlighting its potential as a neuroprotective agent.

MDA is a marker of lipid peroxidation and oxidative stress, both of which are elevated in neurodegenerative conditions and contribute to neuronal damage [63,64]. The significant reduction in MDA levels following SETL treatment indicates that the drug has antioxidant properties, reducing oxidative damage to neuronal cells. Oxidative stress is a major driver of neurodegeneration, and therapies that reduce oxidative damage have been shown to slow the progression of diseases such as AD [65,66]. By decreasing MDA levels, SETL may protect against oxidative damage and help maintain cellular function in the brain. This is particularly relevant in the context of LPS-induced neurotoxicity, where oxidative stress plays a major role in driving neuronal damage. The antioxidant properties of SETL, as evidenced by the reduction in MDA, provide yet another mechanism by which it could protect against neurotoxicity and neurodegeneration.

## 4. Materials and Methods

### 4.1. Dataset

The compounds with known biological activities were obtained from the ChEMBL [67] (https://www.ebi.ac.uk/chembl, accessed on 10 April 2023) database using the UniProt [68] IDs P22303 (https://www.uniprot.org/uniprotkb/P22303/entry, accessed on 10 April 2023) and P353542 (https://www.uniprot.org/uniprotkb/P353542/entry, accessed on 10 April 2023) for AChE and COX-2, respectively. Since the UniProt IDs used for data retrieval correspond to human proteins, only molecules reported for the inhibition of human proteins were considered for model building.

### 4.2. Molecular Descriptors

Molecular descriptors were calculated using RDKit (https://www.rdkit.org, accessed on 23 May 2023), a cheminformatics toolkit. The toolkit provides a range of 1D, 2D, and 3D descriptors, including molecular weight, LogP, topological polar surface area (TPSA), the number of rotatable bonds, hydrogen bond donors and acceptors, and aromatic ring count. These descriptors offer comprehensive insights into the physicochemical properties, structural features, and potential reactivity of the compounds, facilitating the prediction of their biological activities [69].

### 4.3. Machine Learning Models

In this work, three widely used ML algorithms were used to identify dual inhibitors of AChE and COX-2. The algorithms RF, GB and XGB are widely used and highly effective machine learning techniques, each with unique strengths suited to various predictive modeling tasks. RF operates by constructing multiple decision trees and aggregating their predictions to achieve a consensus. Each tree in the forest is built through a series of binary splits that categorize data based on feature values, providing robustness and interpretability, especially for high-dimensional datasets. However, training RFs can be resource-intensive, particularly with large volumes of data [70]. GB improves model performance by sequentially adding decision trees, each designed to correct the errors made by its predecessors. This iterative process enhances the model’s ability to capture complex patterns and relationships, making it versatile and powerful. Nevertheless, it can be computationally demanding, requiring careful parameter tuning [71]. XGB, an optimized variant of GB, further enhances performance through more efficient algorithms and regularization techniques, significantly accelerating training times while improving predictive accuracy. XGB’s advanced capabilities make it highly suitable for handling large and complex datasets [34]. Each of these models has its own set of advantages and challenges, making them suitable for different types of predictive tasks.

### 4.4. Model Validation

Model evaluation is essential for assessing the performance and reliability of machine learning models. Common techniques include cross-validation, where the dataset is partitioned into multiple folds to train and validate the model iteratively, providing a robust estimate of its performance [72]. The confusion matrix offers a detailed view of the classification outcomes, including true positives, false positives, true negatives, and false negatives, which helps in calculating precision, recall, and the F1 score [73]. Accuracy provides a general measure of correctness but may be misleading in imbalanced datasets. Precision and recall are crucial for evaluating performance in such cases, with precision focusing on the correctness of positive predictions and recall assessing the model’s ability to capture all actual positives. The ROC curve and its Area Under the Curve (AUC) offer insights into the model’s ability to distinguish between classes, while metrics such as Mean Squared Error (MSE) and Root Mean Squared Error (RMSE) are used for regression tasks to measure prediction errors [74,75]. The Mean Absolute Error (MAE) provides an alternative to the MSE, offering a less sensitive measure of prediction error [76]. R-squared quantifies the proportion of variance explained by the model, and Log-Loss evaluates the performance of probabilistic classifiers [75,77]. Together, these evaluation methods provide a comprehensive understanding of a model’s effectiveness and guide improvements in predictive performance. In this work, we have used the sensitivity, specificity, precision, overall accuracy, and F1 score for evaluating the performance of the models considered in this study [78,79].

### 4.5. Molecular Docking

Molecular docking simulations were carried out using AutoDock Vina 1.1.2 (AutoDock Vina, CA, USA) to explore the binding interactions of SETL [80]. The target protein structures used were 4EY7 for AChE, 5IKR for COX-2, 1Q4L for GSK-3β, 4I0G for BACE-1, and 1GFW for caspase-3. The preparation of the input files was managed through AutoDock Tools, which is included with MGL Tools (version 1.5.6, La Jolla, CA, USA) [81]. The protein structures were sourced from the Protein Data Bank (PDB), while the three-dimensional structures of the US-FDA approved drugs were obtained from the ZINC15 database. Pre-docking preparation involved removing heteroatoms, water molecules, and any additional chains from the proteins; adding polar hydrogens; filling in missing atoms; and applying Kollman charges. The proteins were then converted to pdbqt format. For SETL, post-minimization in the universal force field was performed, including the definition of torsion angles and the addition of Gasteiger charges, followed by conversion to pdbqt format. Docking grids were set with the small molecule positioned at the grid center. Configuration files were generated to specify receptor details, ligand information, grid box dimensions, and coordinates. The docking results were reported in kcal/mol, and binding modes were analyzed based on the top three low-energy conformations. The interaction with key amino acids was examined for further discussion.

### 4.6. Animal Study

Sertraline hydrochloride and lipopolysaccharide (LPS) from *Escherichia coli* (O111) were obtained from Sigma-Aldrich Co. (St. Louis, MO, USA) and prepared in normal saline (0.09% *w*/*v*). Twenty-four adult male albino rats were sourced from the College of Pharmacy’s animal facilities at Qassim University, KSA, for this study. These rats, weighing between 150 and 170 g and aged 11 to 12 weeks, were divided into four groups, with each group consisting of six animals. The rats were housed three per cage and kept under controlled conditions of a 12 h light/dark cycle. Food and water were available to them at all times. Before the administration of any drug treatments, the animals were given a week to acclimate to the laboratory environment. The Health Research Ethics Committee of the Deanship of Scientific Research at Qassim University approved this study (Research No. 23-61-08, Grant No. 2023-SDG-1-HMSRC-36196). The control group (Group 1) received oral doses of normal saline (0.1 mL/100 mg) daily from day 1 to day 30, alongside intraperitoneal (i.p.) injections of saline (1 mg/kg) on days 27 through 30. Group 2 (SETL) was administered oral SETL at 20 mg/kg daily for 30 days, paired with i.p. saline injections on the same schedule as the control group. Group 3 (LPS) received saline orally from day 1 to day 30 but was injected with LPS (1 mg/kg) i.p. from day 27 to day 30. Group 4 (SETL+ LPS) was treated with oral SETL at 20 mg/kg for 30 days while also receiving i.p. LPS injections on days 27 to 30. These dose regimens for SETL [82,83] and LPS were selected based on previous studies [37,84].

### 4.7. Preparation of Brain Homogenates

On the 30th day of treatment, all animals were euthanized by cervical decapitation under anesthesia, induced via intraperitoneal administration of ketamine (100 mg/kg) and xylazine (10 mg/kg). The brains were then carefully extracted, cut into small pieces, and rinsed thoroughly with ice-cold phosphate-buffered saline (PBS, pH 7.4) to remove any remaining blood. The tissue samples were subsequently weighed and homogenized in PBS at a ratio of 1 g of tissue to 9 milliliters of PBS, using a glass homogenizer on ice. The homogenates were then utilized for ELISA assays to detect the levels of AChE, BACE-1, GSK-3β, COX-2, caspase-3, and MDA.

### 4.8. Biochemical Assays

The levels of AChE, BACE-1, GSK-3β, COX-2, caspase-3, and MDA were measured using specific ELISA kits from MyBioSource (MBS728879; MyBioSources Inc., San Diego, CA, USA), following the manufacturer’s instructions. To summarize, after preparing all necessary reagents, standards, and samples, 100 µL of either the standard solution or sample was added to wells pre-coated with the specific antibody and incubated for 1 h at 37 °C. After incubation, the wells were aspirated, and 100 µL of detection reagent A was added, followed by a 1 h incubation at 37 °C. The plates were then washed three times. Next, 100 µL of detection reagent B was added, incubated for 30 min at 37 °C, and then washed five times. Subsequently, 90 µL of substrate solution was added to each well, followed by 50 µL of stop solution. The optical density was immediately measured at 450 nm using a BioTek microplate reader (BioTek Instruments, Winooski, VT, USA).

## 5. Conclusions

In summary, ML and molecular docking revealed the potential of SETL as an MTDL for AChE, COX-2, GSK-3β, BACE-1, and caspase-3. The molecular docking studies showed the significant binding potential of SETL against the abovementioned proteins. Further, the results from the biochemical analyses of brain samples from the LPS- and SETL-treated rats suggest that SETL may exert multifaceted neuroprotective effects in the LPS-induced neurotoxicity model, affecting key pathways involved in cholinergic function, amyloidogenesis, inflammation, apoptosis, and oxidative stress. The significant reductions in AChE, BACE-1, GSK-3β, COX-2, caspase-3, and MDA levels highlight SETL’s potential to protect against neurodegeneration through its anti-inflammatory, anti-apoptotic, and antioxidant properties. These findings provide a strong rationale for the further investigation of SETL as a therapeutic agent in neurodegenerative diseases, particularly AD, where its ability to modulate multiple pathological processes could offer significant clinical benefits. Despite the encouraging results, the key limitations of this work are the absence of molecular dynamics simulation studies and in vitro evaluation of SETL against the target enzymes. The molecular dynamics simulation studies could have provided a more detailed kinetic analysis of binding interactions, while in vitro evaluations would have confirmed the biological targets for SETL.

## Figures and Tables

**Table 1:** The performance metrics of three machine learning models used in this study.

AChE	TP	FP	TN	FN	Sensitivity	Specificity	Precision	F1-Score	Accuracy
RF	724	51	711	23	0.96	0.93	0.93	0.95	0.95
GB	727	57	705	20	0.97	0.92	0.92	0.95	0.95
XGBoost	728	63	699	19	0.97	0.92	0.92	0.94	0.94
**COX-2**									
RF	538	50	506	33	0.94	0.91	0.91	0.93	0.92
GB	542	63	493	29	0.95	0.89	0.88	0.92	0.92
XGBoost	549	70	486	20	0.96	0.88	0.87	0.92	0.92

## Data Availability

The data presented in this study are available from the corresponding author upon reasonable request. The data are not publicly available due to privacy issues.

## Supplementary Materials

The following supporting information can be downloaded at: https://www.mdpi.com/article/10.3390/molecules29225354/s1, Table S1: Settings/hyper parameters and performance metrics of the three machine learning models used in the study; Table S2: Comparison of the binding affinity of co-crystallized ligand with SETL; Figure S1. Overlapping of docked ligand and co-crystallized ligand; Figure S2. Overlapping of SETL and co-crystallized ligand; Figure S3: Hydrogen and hydrophobic interactions of co-crystallized ligand.
