# Supplementary material for: Sertraline as a Multi-Target Modulator of AChE, COX-2, BACE-1, and GSK-3β: Computational and In Vivo Studies
Source: Molecules. 2024 Nov 14;29(22):5354. doi: 10.3390/molecules29225354 (PMC11596569; doi:10.3390/molecules29225354)
Supplement: Supplementary file 1 [file molecules-29-05354-s001.zip › molecules-3269627-supplementary.pdf]

Supporting Information for **“Sertraline as a Multi-Target modulator of AChE, COX-2, BACE-1, and GSK-3: Computational and In Vivo Studies”**

Table S1. Settings/hyper parameters and performance metrics of the three machine learning models used in the study.

AChE	**Settings/Hyperparameter**	TP	FP	TN	FN	Sensitivity	Specificity	Precision	F1-Score	Accuracy
RF	n_estimators: 400, min_samples_split: 2, min_samples_leaf: 2, max_features: sqrt, max_depth: None, bootstrap: False	724	51	711	23	0.96	0.93	0.93	0.95	0.95
GB	subsample: 1.0, n_estimators: 400, min_samples_split: 10, min_samples_leaf: 2, max_features: sqrt, max_depth: 8, learning_rate: 0.2	727	57	705	20	0.97	0.92	0.92	0.95	0.95
XGB	colsample_bytree: 0.6, gamma: 0.1, learning_rate: 0.05, max_depth: 10, min_child_weight: 3, n_estimators: 300, reg_alpha: 0.01, reg_lambda: 0.1, subsample: 1.0	728	63	699	19	0.97	0.92	0.92	0.94	0.94
COX-2										
RF	n_estimators: 400, min_samples_split: 2, min_samples_leaf: 2, max_features: sqrt, max_depth: None, bootstrap: False	538	50	506	33	0.94	0.91	0.91	0.93	0.92
GB	n_estimators: 500, min_samples_split: 5, min_samples_leaf: 2, max_features: log2, max_depth: 7, learning_rate: 0.2	542	63	493	29	0.95	0.89	0.88	0.92	0.92
XGB	colsample_bytree: 1.0, gamma: 0.5, learning_rate: 0.1, max_depth: 5, min_child_weight: 2, n_estimators: 500, reg_alpha: 0, reg_lambda: 0.01, subsample: 0.8	549	70	486	20	0.96	0.88	0.87	0.92	0.92

Table S2. Comparison of the binding affinity Co-Crystallized ligand with the SETL. The binding affinity has been calculated as the free energy (kcal/mol).

Sr. No.	Ligands	AChE	COX-2	GSK-3	BACE-1	CASPASE-3
1	Co-CL	12.1	9.1	9.2	7.6	8.8
2	SETL	10.5	8.6	9.2	7.2	8.4

.

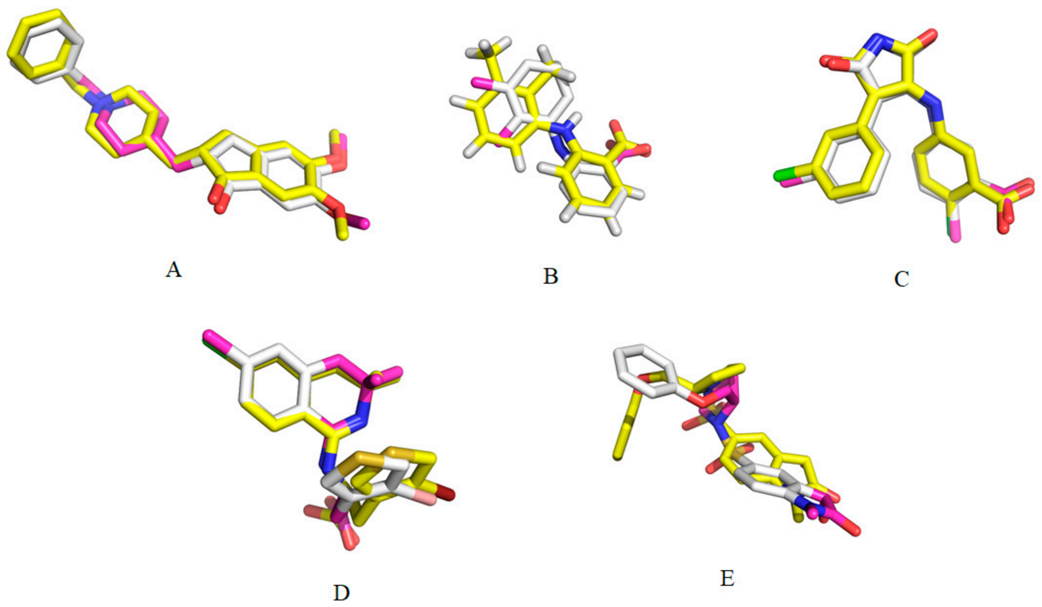

Figure S1. Overlapping of docked ligand (white-pink) and Co-crystallized ligand (yellow). (A) AChE, (B) COX-2, (C) GSK-3β, (D) BACE and (E) caspase-3.

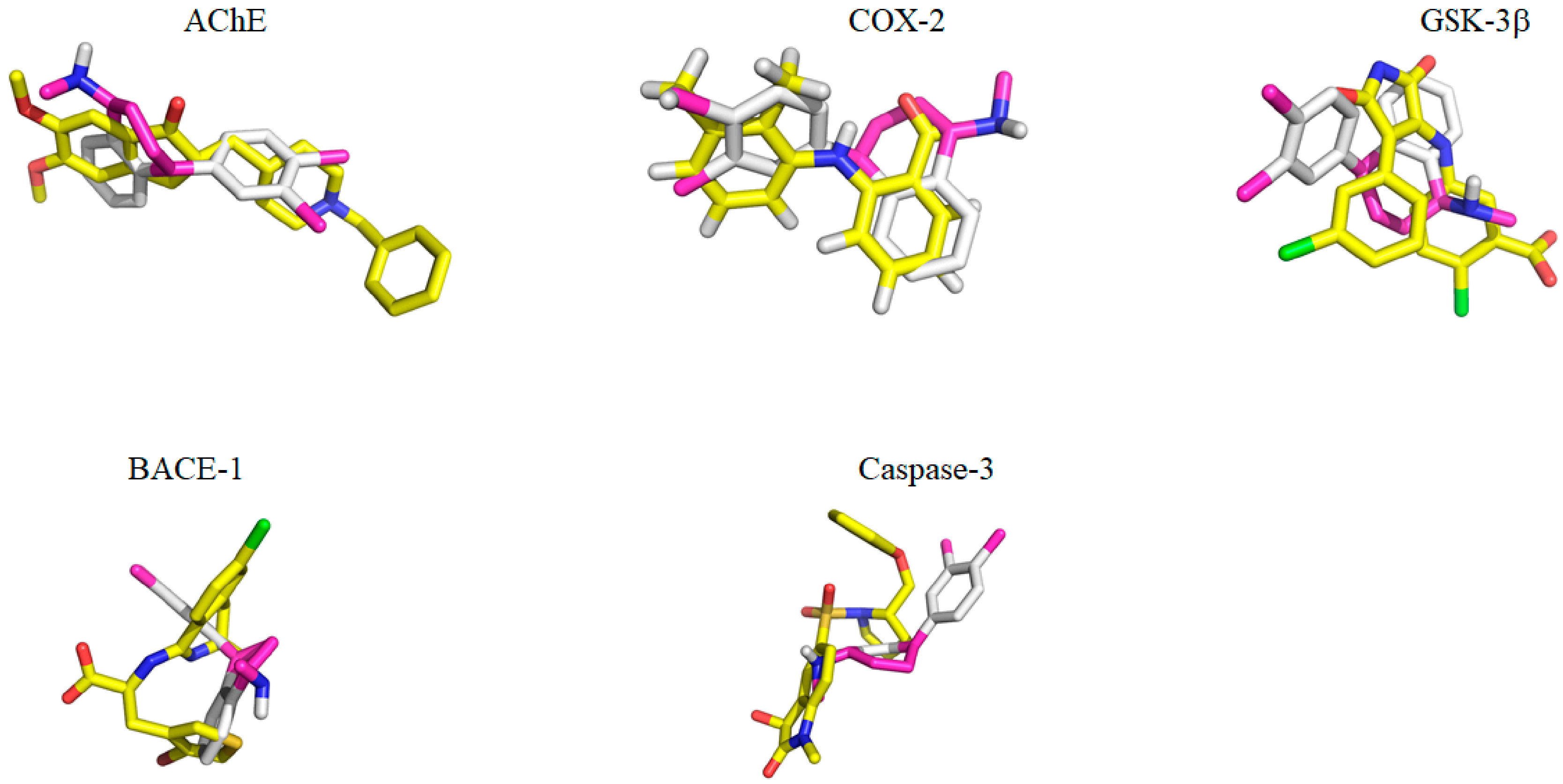

Figure S2. Overlapping of SETL (white-pink) and Co-crystallized ligand (yellow). AChE, COX-2, GSK-3β, BACE-1 and caspase-3.

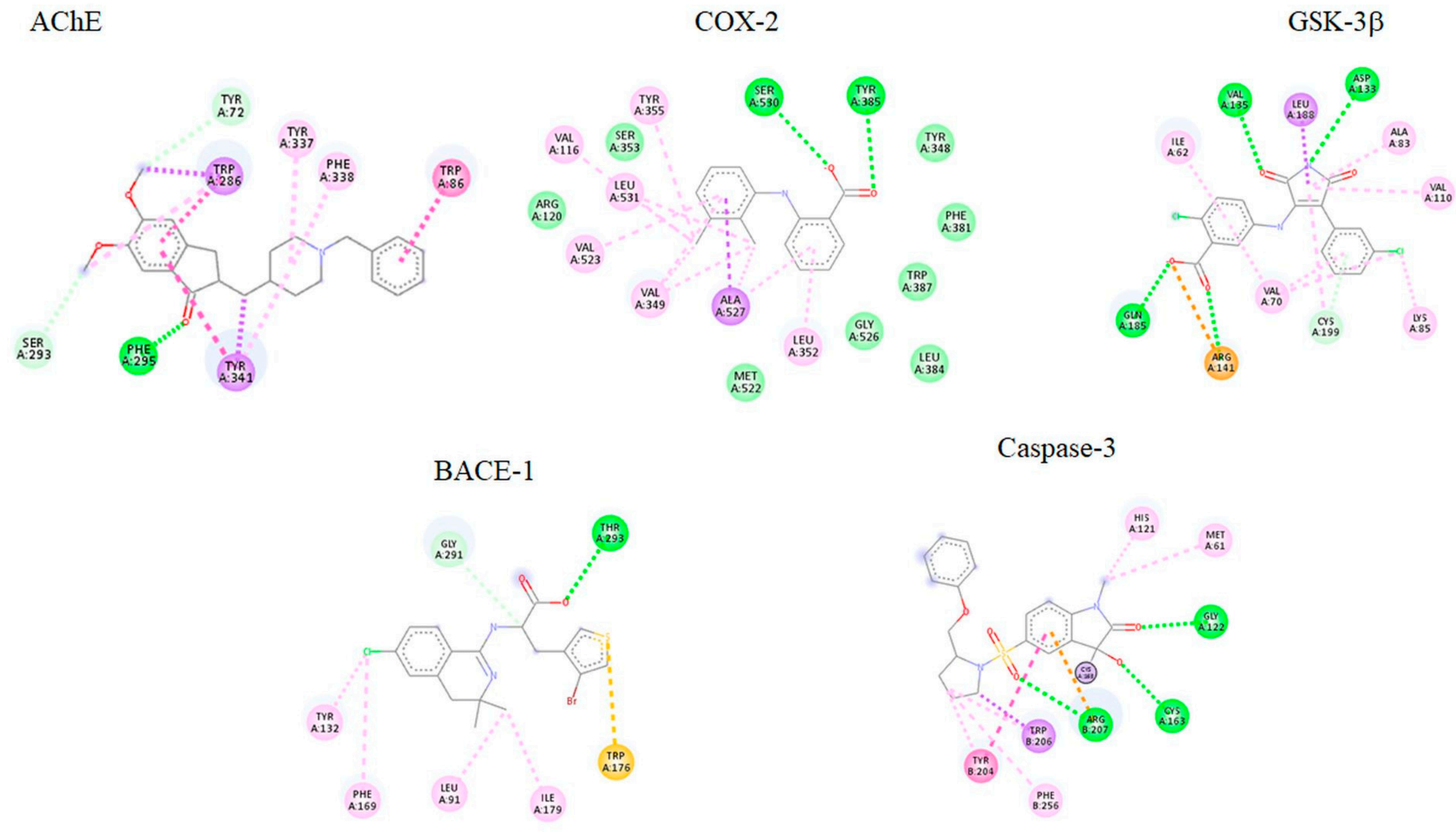

Figure S3. Hydrogen and Hydrophobic interactions of Co-crystallized ligand (AChE, COX-2, GSK-3β, BACE-1 and caspase-3).
